# Chitosan with Sulfonic Groups: A Catalyst for the Esterification of Caprylic Acid with Methanol

**DOI:** 10.3390/polym13223924

**Published:** 2021-11-13

**Authors:** José Castanheiro

**Affiliations:** MED-Instituto Mediterrâneo para a Agricultura, Ambiente e Desenvolvimento, Departamento de Química, Escola de Ciências e Tecnologia, Universidade de Évora, 7000-671 Évora, Portugal; jefc@uevora.pt

**Keywords:** chitosan, heterogeneous catalyst, esterification

## Abstract

Esterification of caprylic acid with methanol was performed over chitosan with sulfonic acid groups, as a catalyst, at 60 °C. The sulfonic acid groups were introduced into chitosan (CH) by using chlorosulfonic acid. Catalysts were characterized by scanning electron microscopy (SEM), elemental analysis, thermogravimetric analysis (TGA), X-ray diffraction (XRD), Fourier transform infrared spectroscopy (FTIR), and acid–base titration. Catalytic activity increased with the amount of sulfonic acid groups present on chitosan. The 4–CH–SO_3_H catalyst (chitosan with sulfonic acid groups—sample 4 prepared) showed the highest activity of all materials. The esterification of caprylic acid with methanol was optimized using a 4–CH–SO_3_H catalyst. Under optimized reaction conditions, it was found that, at 60 °C, with 0.2 g of catalyst loading and with a molar ratio methanol to caprylic acid equal 1:95, a caprylic acid conversion of about 83%, after 4 h could be obtained. Catalytic stability of the 4–CH–SO_3_H material was evaluated through consecutive batch runs. After the second batch, the catalytic activity stabilized.

## 1. Introduction

Biodiesel is a mixture of fatty acid methyl ester. It can be obtained by esterification of fatty acids with an alcohol (methanol, ethanol) and by transesterification of triglycerides with an alcohol (methanol, ethanol, propanol, or butanol) [1,2,3,4]. The transesterification of triglycerides has been performed using homogenous catalysts, such as NaOH or KOH. However, when the amount of free fatty acids present in vegetable oil or animal fats is high, the transesterification reaction cannot occur due to the formation of soap. It is very important to reduce the amount of the free fatty acids present in raw material so as to not influence the transesterification reaction. The fatty acids present in vegetable oil or animal fats can be reduced by an earlier reaction (an esterification reaction) between the fatty acids and an alcohol to produce biodiesel. Traditionally, this reaction is carried out in the presence of homogenous catalysts, such as H_2_SO_4_. However, homogenous catalysts have some disadvantages such as being difficult to recover the catalyst from the reaction mixture and its reutilization [1,2,3,4]. To overcome the problems associated with the homogenous catalyst, esterification reactions have been carried out using solid acid materials, which can be easily removed from the mixture and reused [5,6,7,8,9,10,11]. The esterification of palmitic acid with methanol was performed using poly (vinyl alcohol) (PVA) and polystyrene with sulfonic acid groups. The PVA catalysts showed catalytic activity higher than the polystyrene ones [5]. The esterification of oleic acid with ethanol was carried out over zeolite Y prepared from kaolin [6]. An oleic acid conversion of 85% was obtained, under optimal conditions (T = 70 °C, 5 wt.% catalyst loading and 6:1 ethanol to oleic acid molar ratio) [6]. The esterification of oleic acid with methanol was performed using tungstophosphoric acid supported on MCM-48 [7]. After 8 h of reaction, under optimized conditions (molar ratio of acid to alcohol 1:20; m = 0.1 g; T = 60 °C), the oleic acid conversion was 95% [7]. The esterification of palmitic, oleic, and stearic acid with methanol was studied using tungstophosphoric acid immobilized in SBA-15 [8]. The catalytic activity decreased with the increase in carbon chain number [8]. A. Patel et al. [9] studied the esterification of oleic acid with methanol over sulfonated zirconia. It was observed that the yield (%) increased with the rise in the oleic acid/methanol ratio and reached a maximum of 40% at a molar ratio of 1:40. Moreover, the maximum yield (%) was obtained at a temperature of 60 °C. Resende et al. [10] studied the esterification of fatty acids with methanol using clays as catalysts. The conversion of fatty acids decreases when the hydrocarbon chain increases, which can be due to the difficulty of the active sites gaining access to the catalyst [10]. Waste cooking oils (with 27.8 wt.% FFA) were used for biodiesel production, over solid acid catalysts prepared from D-glucose, sucrose, cellulose, and starch. The starch-derived catalyst showed the highest catalytic activity [11].

Chitosan materials were used as heterogeneous catalysts for different applications, such as aldol condensation, linear aldehyde self-condensation, Henry reaction, and Michael addition [12,13,14,15]. Chitosan with sulfonic acid groups was used as a heterogeneous catalyst on the synthesis of imidazole derivatives, which allowed an easy recovery of the catalyst from the reaction mixture. The reactions were carried out under microwave irradiation [16].

The esterification of palmitic acid over chitosan was studied with sulfonic acid groups [17]. These acid groups were introduced in a chitosan by reaction between natural polymer and sulfosuccinic acid (as a cross-linker). Additionally, sulfosuccinic acid supported on chitosan was used as a heterogenous catalyst in the esterification of levulinic acid with ethanol [18].

Sulfochitosan-coated Fe_3_O_4_ magnetic nanoparticles have been used as a heterogeneous catalyst for the synthesis of 2-amino-4H-chromen-4-yl phosphonates [19].

The esterification of caprylic acid with ethanol was studied over SO_4_^2−^/Fe_x_Al_1−x_PO_4_ [20]. The authors observed that the doping of iron could improve the catalytic activity of SO_4_^2−^/AlPO_4_. The catalytic stability of materials doping with iron improves the stability of the SO_4_^2−^/AlPO_4_ catalyst in successive reaction cycles. The esterification of caprylic acid with methanol was carried out over Nb_2_O_5_, as a catalyst. High caprylic acid conversion was obtained after 3 h of reaction [21]. Bosco et al. [22] studied the esterification of caprylic acid with glycerol using sulfated pillared clay, as a catalyst. Under optimized conditions (caprylic acid:glycerol molar ratio 8:1, at T = 423 K and 5 wt.% catalyst), a total glycerol conversion was obtained after 5 h. In these conditions, yields of 27, 43, and 30%, respectively, for monocarpylin, dicaprylin, and tricaprylin were obtained [22]. Moreover, the esterification of caprylic acid with methanol was carried out over WTS3M (water treatment sludge, with an acid treatment, H_2_SO_4_, 3M) [23]. After 3 h, a caprylic conversion of 98% was obtained, under optimized conditions (methanol: caprylic acid molar ratio of 15:1, 5 wt.% of WTS3M, 100 °C) [23].

This work aims to study the esterification of caprylic acid with methanol, as a model reaction, in the presence of chitosan with –SO_3_H groups. The sulfonic acid groups were introduced into chitosan by a reaction with chlorosulfonic acid. This methodology for preparing chitosan with sulfonic acid groups has an advantage over chitosan prepared with sulfosuccinic acid [17,18], as the number of active sites becomes independent of the degree of crosslinking of the polymer. When sulfosuccinic acid is used to prepare the catalysts [17,18], the amount of sulfonic acid groups increases with the degree of crosslinking, but restrictions on the movement of molecules may increase [17]. To optimize the caprylic acid conversion, different parameters were studied. In addition, the material with high catalytic activity was reused.

## 2. Materials and Methods

### 2.1. Catalysts Preparation

The introduction of sulfonic acid groups was performed by a reaction between chitosan and chlorosulfonic acid (Sigma-Aldrich, Darmstadt, Germany). Adding 5 g of chitosan (Sigma-Aldrich) to 100 cm^3^ of dichloroethane (Sigma-Aldrich, the mixture was stirred for 1 h, at 25 °C. After this period, an appropriate amount of chlorosulfonic acid (0.004, 0.008, 0.015, 0.023 and 0.030 mol for the catalyst 1–CH–SO_3_H, 2–CH–SO_3_H, 3–CH–SO_3_H, 4–CH–SO_3_H, and 5–CH–SO_3_H, respectively) was added dropwise under stirring for 30 min in an ice-bath. The catalyst was then removed from the mixture by filtration. After this operation, the materials were washed with dichloroethane, and dried at room temperature.

### 2.2. Catalyst Characterization

The swelling degree of materials was performed according to Caetano et al. [17].

Acid capacities of material were determined by acid–base titration, according to Caetano et al. [17].

The FTIR spectra were recorded in a Perkin Elmer Spectrum 100 FTIR spectrometer (PerkinElmer, Shelton, WA, USA).

CHNS Elemental Analyzer 1112 series (Thermo Finnigan, San Jose, CA, USA) was used to determine the Sulphur.

NETZSCH STA 449F3 thermogravimetric analyzer (NETZSCH, Selb, Germany) was used to study the thermal stability CH and 4–CH-SO_3_H.

Zeiss Auriga equipment (Zeiss, Oberkochen, Germany) was used to obtain SEM images.

X-ray diffraction was recorded in a RIGAKU Miniflex II (RIGAKU, Neu-Isenburg, Germany).

### 2.3. Catalytic Experiments

The catalytic tests were performed using 8 mmol of caprylic acid, 30 mL of methanol, and 0.2 g of catalyst, at 60 °C.

Stability tests of the 4–CH–SO_3_H material were performed running five consecutive experiments. Between reactions, the material was washed with methanol and dried during 24 h, at 80 °C.

During the reaction, some samples were picked up. Undecane was used as the internal standard. The samples were analyzed using a GC (Hewlett Packard instrument (Hewlett-Packard Company, Palo Alto, CA, USA)).

### 2.4. Determination of Kinetic Constants, k (min^−1^)

To determination of the value of kinetic constants, k (min^−1^), Equation (1), was applied according to the Pessoa Junior et al. [23]:

(1) $$\ln\left(1-X_{t}\right)=k\cdot t$$

where *X_t_* is the conversion of caprylic acid at time *t*

## 3. Results and Discussion

**Figure 1:**
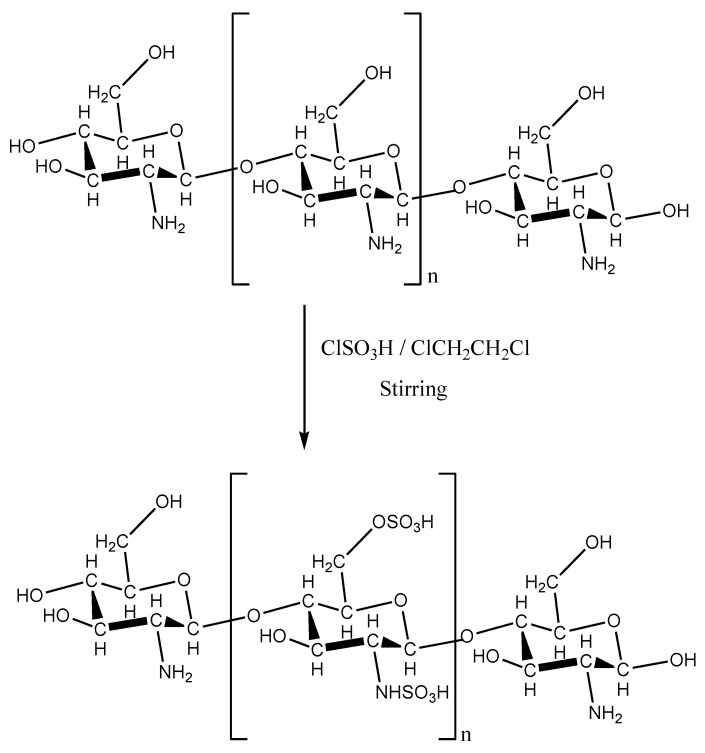
Scheme of the preparation of chitosan with sulfonic acid groups (R = –H or –COCH_3_).

Figure 1 shows the scheme of preparation of chitosan with sulfonic acid groups.

Table 1 shows the characteristics of materials. It was observed that the acidity of the chitosan with –SO_3_H groups enlarged with the increase in the number of sulfonic acid groups introduced on the chitosan matrix. Table 1 also shows the sulfur amount in the chitosan with sulfonic acid groups, which was determined via elemental analysis. Similar results for the acid capacities, obtained by acid–base titration and those obtained by elemental analysis, were apparent [20,24].

The swelling degree of materials, for methanol and water, reduced when the sulfonic acid groups were increased (Table 1). This behavior could be due to the decrease in free volume [20,24].

**Figure 2:**
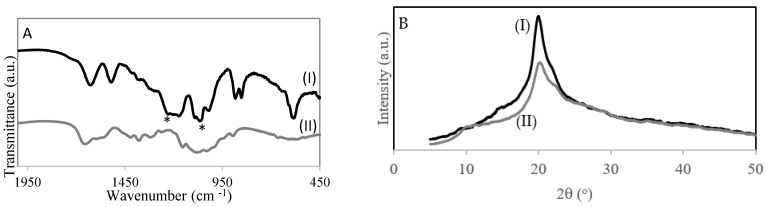
(**A**) FT-IR spectra of catalysts: (I) 4-CH-SO_3_H; (II) chitosan. (**B**) XRD of (I) chitosan and (II) 4–CH–SO_3_H catalyst.

Figure 2A shows the FT-IR spectra of chitosan (Figure 2AI) and chitosan with sulfonic acid groups (Figure 2AII). The spectrum of the 4–CH–SO_3_H sample (Figure 2AII) shows a band at 1226 cm^−1^ corresponding to –S = O stretching bands of –SO_3_H in –O–SO_3_H and a band at 1064 cm^−1^ –S = O stretching bands of −SO_3_H in NH−SO_3_H groups, which suggests the introduction of the sulfonic acid group onto chitosan [20].

Figure 2B shows the X-ray diffraction patterns of chitosan and the 4–CH–SO_3_H catalyst. The pattern of chitosan has a characteristic crystalline peak at 2θ degrees near to 20° [18]. The 4–CH–SO_3_H catalyst also has a peak at 2θ degrees of 20°, which is similar to that of chitosan. However, the intensity of the 4–CH–SO_3_H catalyst peak is lower than that of chitosan. A decrease in the degree of crystallinity was detected. This result could be due to the introduction of sulfonic acid groups on the chitosan structure [20].

**Figure 3:**
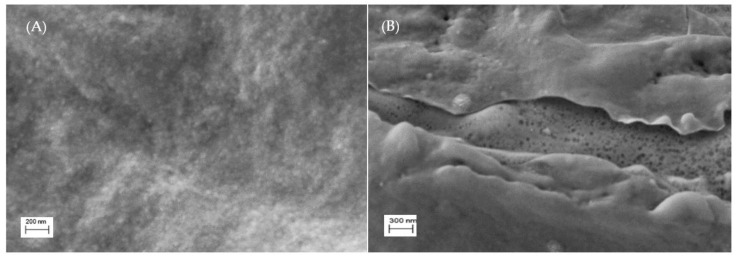
SEM morphology images of the surface of the chitosan (**A**) and 4–CH–SO_3_H material (**B**).

The surface SEM micrographs for chitosan (CH) and chitosan with sulfonic acid (4–CH–SO_3_H sample) are shown in Figure 3. It can be seen that the 4–CH–SO_3_H catalyst shows voids and cracks in its structure (Figure 3B) while the chitosan matrix (without sulfonic acid groups) exhibits a void-free dense structure (Figure 3A). The presence of some voids and cracks in the 4–CH–SO_3_H material could improve the access of methanol and/or caprylic acid to the active sites of the catalyst [16,17].

**Figure 4:**
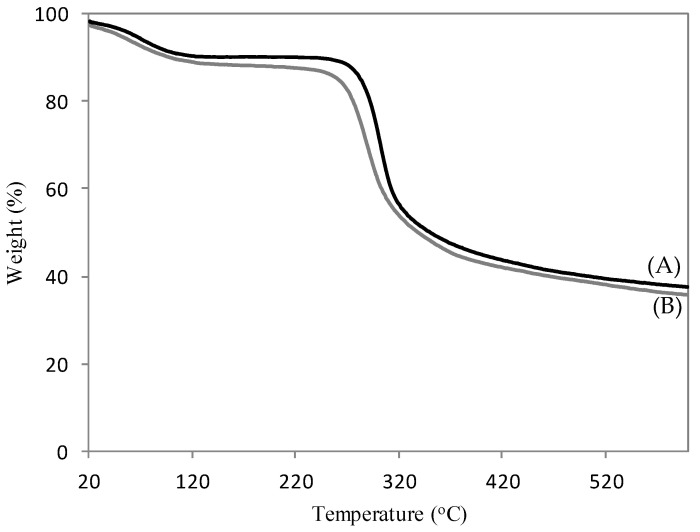
TG curves of the chitosan (**A**) and the 4–CH–SO_3_H catalyst (**B**).

Figure 4 shows the TG analysis of CH (Figure 4A) and 4–CH–SO_3_H samples (Figure 4B). Two major stages of degradation at 90–200 °C and 200–450 °C were observed. The first weight loss is attributed to the desorption of physically absorbed water molecules and acetic acid. The second weight loss is attributed to the thermal degradation of chitosan with sulfonic acid groups [25]. As the reaction studied is performed at 60 °C, it is possible conclude that the 4–CH–SO_3_H sample is thermally stable.

### 3.1. Catalytic Experiments

The esterification of caprylic acid (octanoic acid) with methanol was performed using chitosan with sulfonic acid groups, as heterogeneous catalysts, at 60 °C, obtaining methyl octanoate plus water.

**Figure 5:**
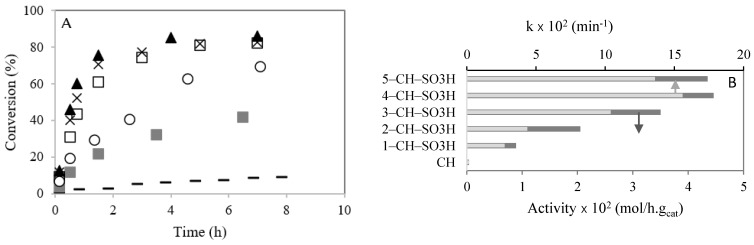
Esterification of caprylic acid with methanol in the presence of chitosan with SO_3_H, at 60 °C: (**A**) conversion (%) versus time (h). (–) CH; (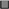) 1–CH–SO_3_H; (◯) 2-CH–SO_3_H; (☐) 3–CH–H; (▲) 4–CH–SO_3_H; (×) 5–CH–SO_3_H; (**B**) initial activities taken as the maximum observed reaction rate and k (min^−1^). Reaction conditions: catalyst amount (0.2 g); temperature (60 °C), molar ratio caprylic acid to methanol (1:95).

Figure 5A shows the conversion (%) of caprylic acid versus time (h). After 90 min of reaction, the caprylic acid conversion was 22, 30, 61, 75 and 72% for the 1–CH–SO_3_H, 2–CH–SO_3_H, 3–CH–SO_3_H, 4–CH–SO_3_H, and 5–CH–SO_3_H materials, respectively.

The initial catalytic activities of chitosan with sulfonic acid in the esterification of caprylic acid with methanol are compared in Figure 5B (dark bars). It was observed that the initial activity of chitosan with sulfonic acid groups increased with the sulfonic group numbers. This behavior could be due to the increase in value of the acidity (Table 1). However, when the sulfonic acid amount raised from the 4–CH–SO_3_H catalyst to the 5–CH–SO_3_H catalyst, there was no significant increase in acidity (Table 1) and caprylic acid conversion. Previously, Singh et al. [6] studied the esterification of oleic acid with methanol using heteropolyacids immobilized on MCM-48 as catalysts. The authors ascertained that the catalytic activity increased with the number of active sites [6].

The kinetic constants (k), calculated by Equation (1) after 90 min of reaction, are compared in Figure 5B (light bars). It is observed that k increased with the amount of −SO_3_H present on chitosan, which can be explained by the increase in acidity (Table 1). However, a slight decrease in k is observed from the catalyst sample 4–CH–SO_3_H to the catalyst 5-CH-SO_3_H.

Consequently, the 4–CH–SO_3_H material was used for our full study.

### 3.2. Effect of Catalyst Loading

**Figure 6:**
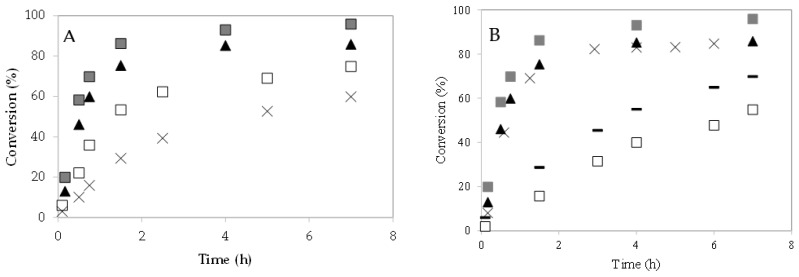
Esterification of caprylic acid with methanol over 4–CH–SO_3_H catalyst. Effect of catalyst loading: (**A**) conversion (%) versus time (h): (×) m = 0.08 g; (☐) m = 0.14 g; (▲) m = 0.2 g; (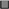) m = 0.3 g;. (**B**) effect of molar ratio of alcohol to caprylic acid. Conversion (%) versus time (h): (☐) 1:15 (m = 0.2 g); (×) 1:63 (m = 0.2 g); (▲) 1:95 (m = 0.2 g); (−) 1:15 (m = 0.3 g); (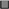) 1:95 (m = 0.3 g).

The effect of the 4–CH–SO_3_H catalyst loading on caprylic acid conversion was studied at 60 °C (Figure 6A). The increase in caprylic acid conversion with the 4–CH–SO_3_H concentration could be due to an increase in the number and availability of active sites [8,9,23]. With a m = 0.3 g of catalyst, a caprylic acid conversion of 87% was obtained after 90 min of reaction.

### 3.3. Effect of Molar Ratio Alcohol to Caprylic Acid

The esterification reactions were performed using an excess of alcohol to favor the forward reaction, since these reactions are reversible. To study the effect of molar ratio caprylic acid to methanol on reaction, different experiments were carried out using 1:15, 1:63, and 1:95. The 4–CH–SO_3_H catalyst amount (0.2 g) and the reaction temperature (60 °C) were kept constant. It was observed that when the molar ratio increases from 1:15–1:63, the conversion of caprylic acid increases, at a fixed time (Figure 6B). However, when the molar ratio value increases from 1:63–1:95, an increase in conversion was not observed. Similar results were also noted by S. Singh et al. [6]. Additional experiments were carried out with m = 0.3 g of catalyst. When the amount of catalyst increased from m = 0.2 g to m = 0.3 g, the conversion of caprylic acid also increased.

### 3.4. Effect of the Alcohol Nature and Temperature

**Figure 7:**
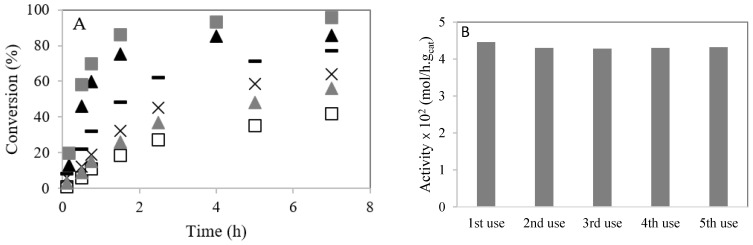
Esterification of caprylic acid with methanol or ethanol over 4–CH–SO_3_H catalyst: (**A**) effect of temperature. Conversion (%) versus time (h): (▲) reaction with methanol at 60 °C (m = 0.2 g); (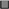) reaction with methanol at 60 °C (m = 0.3 g); (☐) reaction with ethanol at 60 °C (m = 0.2 g); (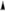) reaction with ethanol at 60 °C (m = 0.3 g); (×) reaction with ethanol at 80 °C (m = 0.2 g); (−) reaction with ethanol at 80 °C (m = 0.3 g); (**B**) stability studies of the 4–CH–SO_3_H catalyst on esterification of caprylic acid with methanol.

Figure 7A shows the conversion of caprylic acid with methanol and ethanol at 60 °C. The loading of 4–CH–SO_3_H catalyst (m = 0.2 g) and the temperature (T = 60 °C) were kept constant. A decrease in the caprylic acid conversion is observed when ethanol is used compared with the experiment using methanol. This result can be explained by ethyl nucleophile being less reactive than methyl nucleophile [26,27]. Likewise, steric hindrance increases with molecular size [21,23]. When the reaction is performed using ethanol at 80 °C, an increase in the reaction rate is observed. The conversion of caprylic acid is increased when the amount of catalyst increases from m = 0.2 g to m = 0.3 g.

### 3.5. Catalyst Stability and Reusability

The 4–CH–SO_3_H material was reused for consecutive reactions. A stabilization of the initial activity after the second use was observed (Figure 7B). A small amount of –SO_3_H groups cannot be linked to the chitosan. The loading of acid sites of 4–CH–SO_3_H material was determined by an acid–base titration and elemental sulfur analysis. After five cycles, the amount of S decreased slightly (Table 1).

Table 2 shows the comparation of caprylic acid conversion using chitosan with sulfonic acid groups, as catalysts, with other catalysts reported in the literature. The 4–CH–SO_3_H catalyst shows a high catalytic activity. Chitosan is a biopolymer that can be used as a heterogenous catalyst for the esterification of caprylic acid with methanol.

## 4. Conclusions

The esterification of caprylic acid was carried out in the presence of chitosan with sulfonic acid at 60 °C. The catalytic activity of polymeric of materials increased with the amount of the sulfonic acid catalyst until a maximum was reached, which was obtained with the 4–CH–SO_3_H sample. Under optimized reaction conditions (m = 0.2 g of 4–CH–SO_3_H catalysts, T = 60 °C, and molar ratio caprylic acid to methanol 1:95), a 75% of caprylic acid conversion was obtained. The esterification of caprylic acid with methanol led to higher conversion when compared with ethanol at the same reaction temperature.

The catalytic stability of the 4–CH–SO_3_H material was evaluated through different consecutive batch runs with the same catalyst. The activity stabilized after the second batch.

## Figures and Tables

**Table 1:** Characterization of chitosan and chitosan with sulfonic acid groups.

Sample	Acid Capacities		Methanol Swelling ^c^ (%)	Water Swelling ^d^ (%)
	Titration ^a^	Scontent ^b^		
CH	0.40	−	0.93	1.61
1–CH–SO_3_H	1.28	1.30	0.063	1.53
2–CH–SO_3_H	2.05	2.09	0.032	1.44
3–CH–SO_3_H	3.09	3.11	0.022	1.29
4–CH–SO_3_H	3.19 3.17 ^f^	3.21 3.19 ^f^	0.015 (0.007 ^e^)	1.09
5–CH–SO_3_H	3.25	3.27	0.009	0.77

(^a^) Amount of Brönsted acid sites was determined by acid–base titration (mmol/g). (^b^) Sulphur molar content determined by elemental analysis (mmol/g). (^c^) Methanol swelling was measured by immersing dried pieces catalysts in methanol, at 60 °C. (^d^) Water swelling was measured by immersing dried pieces catalysts in water, at 60 °C. (^e^) Ethanol swelling was measured by immersing dried pieces catalysts in ethanol, at 60 °C. (^f^) After five uses of 4–CH–SO_3_H sample.

**Table 2:** Comparison of caprylic acid conversion with reported catalyst.

Catalyst	Temperature (°C)	Catalyst Amount (g)	Molar Ratio Caprylic Acid: Acohol	% Conversion (Time)	Reference
4–CH–SO_3_H	60	15% wt.	1:95 ^a^	75 (90 min)	Present work
SO_4_^2−^/Fe_0.08_Al_0.92_PO_4_	75	1.5% wt.	1:6 ^b^	90 (240 min)	[19]
Nb_2_O_5_	65	15% wt.	1:14 ^a^	95 (180 min)	[21]
WTS3M	100	5% wt.	1:15 ^a^	98 (180 min)	[23]

(^a^) Methanol. (^b^) Ethanol.

## Data Availability

The data presented in this study are available on request from the corresponding author.
